# An atypical case of bilateral pulmonary hydatid cyst with endo-rupture managed with two-stages uniportal video-assisted thoracoscopic surgery in Palestine: case report

**DOI:** 10.1097/RC9.0000000000000299

**Published:** 2026-02-16

**Authors:** Amal N Abubaker, Sara I Hroub, Amani N Dawada, Laila K Diab, Qais Salah, Yousef Abu Asbeh

**Affiliations:** a Department of Medicine, Palestine Polytechnic University, West Bank, Palestine; b Department of Internal Medicine, Al-Quds University, Jerusalem, PSE; c Department of Thoracic Surgery Unit, Al Ahli Hospital, West Bank, Palestine

**Keywords:** pulmonary hydatidosis, VATS, hydatid cyst, bilateral, capitonnage, Echinococcus granulosus, pulmonary

## Abstract

**Introduction:**

Echinococcosis is a helminthic infection caused by cestodes of the genus *Echinococcus* (e.g., *E. granulosus, E. multilocularis*). Infection is typically acquired by accidental ingestion of Echinococcus eggs, which are shed in the feces of infected dogs. Infection results in cystic lesions in the liver (75% of cases), lungs (15% of cases) and, rarely, other organs.

**Case presentation:**

We report here a case of a bilateral pulmonary hydatid cyst in a 19-year-old female with endo-rupture of the cyst in the right upper lobe. The patient presented with a history of longstanding dry cough, fever, and chest pain.

**Clinical discussion:**

Diagnostic investigations revealed the nature and content of the cysts. Eventually, our patient underwent sequential surgical removal of the cysts using uniportal video-assisted thoracoscopic surgery (VATS). In pulmonary hydatid cyst, the infection usually manifests as a single isolated pulmonary hydatidosis, bilateral pulmonary hydatidosis is unfamiliar presentation.

**Conclusion:**

Bilateral pulmonary hydatidosis is not a common entity, even in endemic areas. VATS has been increasingly used to treat lung cysts with better outcomes.

## Introduction

*Echinococcus granulosus* is a worm that takes dogs as its definitive host and inhabits their intestines; it usually spreads to humans through exposure to dog’s feces^[1]^. Afterward, it resides in one of the most affected organs in the form of a hydatid cyst, such as the liver, lungs, kidneys, or brain^[2]^. The hydatid cyst presents with a wide range of manifestations: it can be completely asymptomatic, or it can present with life-threatening complications. Bilateral pulmonary hydatid cysts are not usual, even in endemic areas, and can present with symptoms such as hemoptysis, dyspnea, and cough for an extended period^[3]^. Rupture of the cysts can be fatal, as it may cause dissemination of the germinal layer, leading to anaphylactic shock. Raising awareness about prevention methods for this worm is crucial. Our case report focuses on a case of a 19-year-old female with bilateral pulmonary hydatid cysts, one in the left lower lung lobe and the other in the right upper lung lobe, complicated by endo-rupture, as she was diagnosed and managed in a Palestinian academic medical center.

**HIGHLIGHTS**

- Bilateral pulmonary hydatidosis is an uncommon entity, even in endemic areas.
- Surgical removal of the cyst is the gold standard therapeutic modality, whether it is a one-stage or two-stage surgery.
- The u-VATS procedure is safe and effective for removing pulmonary hydatid cysts smaller than 15 cm.
- Thoracotomy has lately been substituted by VATS.
- The strengths of our report lie in the documentation of this unusual presentation and the detailed illustration of a modern, minimally invasive surgical approach.
- The high-quality intraoperative notes clearly depict the cyst’s pathology and the key steps of the VATS procedure, providing a valuable reference for thoracic surgeons.

This case report has been reported in line with the SCARE checklist^[4]^.

### Timeline

The patient’s initial complain was prolonged dry cough of 1-month duration. The timeline between the presentation, diagnosis, and therapeutic intervention was between January and February 2023

### Patient information

A 19-year-old female, nursing student from Palestine, living in a rural area, with free past medical and surgical history, presented to the clinic complaining of persistent dry cough that she had for 1 month. The cough started gradually and progressed over time, intensifying upon awakening and around sleep time at night. The patient did not report fever, chest pain, or hemoptysis. Further questioning revealed no known relieving or exacerbating factors. Subsequently, patient developed shortness of breath and left flank pain. The patient had no history of close contact with dogs or other animals and no recent history of respiratory tract infections. There was no earlier family history of the same case. Upon examination, patient appeared well; chest auscultation revealed bilateral crackles in the lungs, the vital signs were stable, and patient was afebrile.

### Clinical findings

General condition: Patient looks well, stable vital signs, afebrile.

Skin, hair, and nails: No pallor, no jaundice, no neck cyanosis.

HEENT/Neck: No congested throat, no neck swelling.

Cardiovascular: Regular heart rate, no murmers.

Respiratory: chest auscultation revealed bilateral crackles in the lungs, decreased air entry on left lower lung, mild tachypnia, no signs of respiratory distress.

Abdominal: Soft lax, not distended.

Neuro/psych exam: COA, GCS = 15/15, power = 5/5 in all limbs, no FND.

Musculoskeletal exam: Free.

### Diagnostic assessment and interpretation:

**Figure 1.**
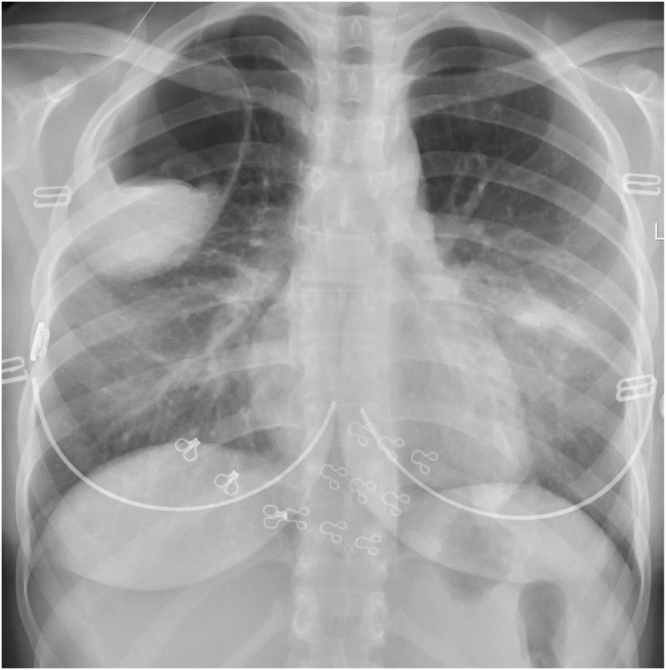
Chest radiograph showing a well-defined cystic lesion in the upper lobe of the right lung.

Chest X-ray was carried out promptly, revealing right upper lung cystic lesion with air-fluid level. (the chest X-ray findings are shown in Fig. 1).

Chest U/S done with conclusion of bilateral well-defined lung hydatid cysts, one in the posterior aspect of the left lower lobe, well-defined anechoic cystic lesion, measuring about 15 × 11 cm, and associated with a small left pleural effusion around it. Another cavitary soft tissue lesion was noted in the posterior right upper lobe, measuring 10 × 6.8 cm, with an air-fluid level indicating an endo-rupture.

**Figure 2.**
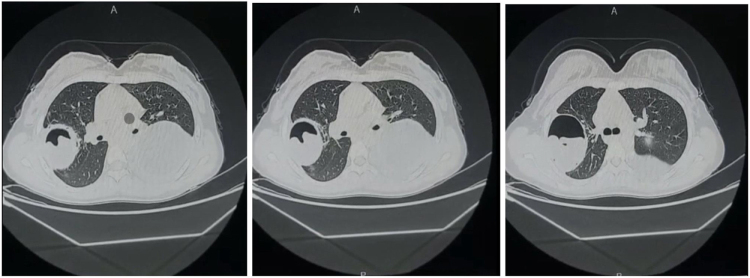
CT of the chest shows bilateral lung cysts, the one at the left side is intact, round, homogeneous, and water-dense with an enhancing rim. The cyst at the right upper lobe is a ruptured cyst; the CT shows air-fluid level with crumpled endo-cyst membrane appearing as floating membrane.

Chest CT scan was performed later (the chest CT findings are shown in Fig. 2).

Abdominal U/S done and no hepatic cystic lesions was found.

Laboratory investigations showed an increased white blood cell count and mildly elevated eosinophil levels. ELISA was done and was positive. Additionally, a nasal swab for COVID-19 was negative. Arterial blood gas tests showed an increased pH of 7.48, decreased partial pressure of carbon dioxide, increased partial pressure of oxygen, and decreased bicarbonate (HCO_3_^−^). Kidney and liver function tests were normal, and serum electrolyte concentrations were within normal limits (the laboratory results were summarized in Table 1).

**Table 1:** Laboratory investigations carried out for the patient upon presentation

Laboratory test	Result	Reference range
White blood cell count	27 × 10^3^/μL	5–10 × 10^3^/μL
Hemoglobin	14.4 mg/dL	12–16 mg/dL
Platelet count	336 × 10^3^/μL	150–450 × 10^3^/μL
Eosinophils differential	4.2%	0–2%
pH	7.48	7.35–7.45
Partial pressure of carbon dioxide	26.5 mmHg	35–48 mmHg
Partial pressure of oxygen	147.2 mmHg	83–108 mmHg
Bicarbonate	20 mEq/L	23–28 mEq/L

### Intervention

Removal of the hydatid cyst was performed in two stages surgery, using bronchoscopy and uniportal video-assisted thoracic surgery (u-VATS).

Surgical management was planned, starting with surgery for the left lung cyst, which was the site of the larger, intact cyst that was at risk of catastrophic pleural rupture, due to its huge size and the presense of pleural effusion that is highly suspecious for leak into pleural space. Surgery for the right lung cyst was planned to follow with an interval of 4 weeks.

The first operation was conducted using bronchoscopy and left u-VATS. Removal of the hydatid cyst was performed using the capitonnage technique. A 4-cm skin incision was made in the left 6th intercostal space, posterior to the posterior axillary line. A mild amount of pleural effusion and a huge hydatid cyst involving the left lower lobe were observed. The cyst contents were aspirated with a needle and then injected with hypertonic saline 0.3% – a scolicidal agent. The saline was left in the cyst for 15 minutes, 10 gauzes soaked with hypertonic saline were applied surrounding the cyst, after which the cyst was opened and the germinal layer was removed, then derofing the cyst using cautery was done, followed by irrigation of the pleural cavity, inflating the lung and identification of four bronchial openings. closure of the bronchial openings was done using 2/0 vicryl inturrupted stitches. Cappetonage and closure of the cyst gradually using multiple interrupted Vicryl stitches. The cyst was closed completely. chest tube was applicated in the apicoposterior positin. No evidence of an air leak was observed. The surgery was completed without complications, and the postoperative course was uneventful. The patient was discharged on the fourth postoperative day after the chest tube was removed.

The subsequent operation was carried out a month later using bronchoscopy and right u-VATS, the hydatid cyst was removed using the capitonnage technique as well. A 5-cm incision was made in the right 6th intercostal space, posterior to the posterior axillary line. A mild amount of pleural effusion and a large hydatid cyst involving the right upper lobe were identified. The cyst was managed and removed in a manner similar to the left side. The patient had an unremarkable operative and postoperative course and was discharged in good condition on the fourth day after surgery.

The patient was started on albendazole upon presentation, and was prescribed a 6-month course of albendazole.

### Follow-up and outcomes

The first operation was completed with good outcomes, the second surgery was done a month later, then the patient was discharged home on the 4th day post-op with an unremarkable operative and postoperative course in good condition.

Forty days after the second operation, the patient was admitted to the surgical ward after complaining of right sided chest pain and difficulty breathing. On auscultation, decreased breath sounds were heard, and a radiologic workup revealed that patient developed severe right pneumothorax. It was managed with a 12-Fr pigtail catheter and observed for 3 days. The drain was removed before patient was discharged. The patient committed to regular follow-up for 1 year, and there was no evidence of disease recurrence or other complications.

## Discussion

Either echinococcosis or a hydatid cyst is a parasitic infectious disease caused by the larval stage of *Echinococcus granulosus*^[5,6]^. Echinococcosis is an endemic disease with a higher prevalence in Russia, the Mediterranean, China, Australia, North and East Africa, and South America^[5]^. There are four pathogenic species out of six, which are *E. granulosus* (the most common, accounting for >95% and responsible for cystic echinococcosis, *Echinococcus shiquicus, Echinococcus multilocularis* (responsible for alveolar echinococcosis), and *Echinococcus felidis*, which does not have zoonotic transmission^[5]^.

Hydatidosis is more common in communities that keep dogs guarding and herding cattle^[7]^. The Echinococcus tapeworms harbor in the small intestines of dogs and other carnivores. Eggs are excreted with feces, and humans are infected incidentally by the ingestion of contaminated water and vegetables^[7,8]^. After the larva released from the eggs, it penetrates the mucosa of the human’s small intestine, they travel through the blood and lymphatic vessels to the liver, lungs, and other organs^[7]^.

The liver is the most affected site, accounting for more than 65% of cases; it is also the most common site in adults. Followed by the lungs, which is 25% of cases and is the most common site in children. Other organs, such as the spleen, heart, bone, kidney, and central nervous system, are rarely involved^[5,8]^. Unilateral pulmonary hydatidosis is more common than bilateral hydatidosis and has a prevalence ranging between 4% and 26.7% in endemic areas^[9]^.

Uncomplicated lung cysts are usually asymptomatic and diagnosed incidentally^[9]^. The slow growth of the cyst handles its late presentation^[5]^. In most cases, pulmonary hydatid cysts present with symptoms such as cough, chest pain, fever, and hemoptysis^[5]^. In our case, the patient presented with a dry cough persisted for 1 month, more pronounced in the morning and at night.

The most common complications of pulmonary hydatidosis are cyst rupture due to secondary infection and pneumothorax. Anaphylactic shock may occur after cyst rupture^[5]^. In our case, the patient presented with a right-side pulmonary cyst rupture but without any progressive symptoms. Another rare complication is hydatid pulmonary embolism, which occurs after the rupture of a cyst^[6]^.

The diagnosis of hydatid cyst disease in endemic areas is established based on history and radiological studies. A positive history is considered with exposure to sheep and dogs^[10,11]^. Radiological investigations and serology are the diagnostic tools of choice for confirmation^[10,12,13]^. In diagnosing pulmonary hydatid disease specifically, chest radiography and computed tomography (CT) scans of the chest are the best methods^[8–10]^. The chest radiograph is used as a first diagnostic tool and is the most valuable modality^[9,11]^, while CT scans are preferred for evaluating complicated cysts and for excluding other differential diagnoses that may resemble pulmonary hydatidosis^[9]^.

Radiological findings of the disease differ based on whether the cyst is complicated or not^[10]^. The classical chest radiographic findings for uncomplicated pulmonary hydatidosis are single or multiple well-defined masses with smooth borders and a homogeneous, rounded shape^[9,10,13]^. The diameter of an intact cyst is variable, ranging between 1 and 20 cm. Single cysts are more common than multiple cysts^[10]^. The size of the cysts varies based on their location; central cysts are smaller than peripherally located cysts, that’s because of the major broncho-vascular structures located centrally preventing the centrally located cysts from expanding^[10,13]^.

Less than 15% of pulmonary hydatid patients have eosinophilia, and other routine blood tests are considered nonspecific^[12,13]^. Increased erythrocyte sedimentation rates and white blood cell counts are also seen; eosinophilia and leukocytosis are more commonly seen in patients with ruptured cysts^[10]^.

In serological investigations, checking for the presence of serum antibodies or circulating antigen receptors serves as a clear, supportive guide in the diagnosis of pulmonary hydatidosis^[13]^. Antibody detection is considered more sensitive than antigen detection^[10]^. Common serological investigations include enzyme-linked immunosorbent assay (ELISA), latex agglutination, indirect hemagglutination test, and immunoblot assay using lentil–lectin-purified glycol proteins^[10,12]^. These tests are more sensitive in cases of hepatic hydatid, reaching 85–95% sensitivity, compared to 50–60% in cases of pulmonary hydatid^[10–13]^.

Surgical removal of the cyst is the therapeutic modality of choice^[8–13]^, as it allows for complete removal of the parasite. It is worth noting that the treating physician should take care not to rupture the cyst intraoperatively to prevent dissemination of the cyst’s contents^[13]^. The entire cyst should be removed to preserve the largest lung parenchyma^[8,9]^. The cyst’s fluid should also be examined to aid in establishing the diagnosis^[12]^. Surgery is not preferable in certain situations, such as patient refusal, pregnancy, multiple cysts with difficult accessibility, multi-organ diseases, and recurrent cysts^[10,11]^. Medical treatment is preferred in selected cases^[10]^, particularly in instances of ruptured cysts, disseminated disease, and patients with surgical contraindications^[13]^.

In cases of bilateral pulmonary hydatidosis, as in our case, management is controversial. It could be conducted as a one-stage surgery or as a two-stage surgery spaced 4–5 weeks apart, starting with the uncomplicated cyst^[9]^. Some authors prefer the two-stage approach, while others believe that one-stage surgery is better, as it is associated with less morbidity, shorter hospital stays, and lower costs^[9]^.

Thoracotomy was the most used procedure for eliminating pulmonary hydatid cysts. While many earlier researchers removed pulmonary hydatid cysts using thoracotomy, mini-thoracotomy, or VATS with two or three ports, early investigations showed that a single incision (uniportal) could be performed, and thoracotomy has lately been substituted by VATS^[14]^. The u-VATS procedure is safe and effective for removing pulmonary hydatid cysts smaller than 15 cm. This technique’s success rate is determined by the experience of cardiothoracic and vascular surgeons, as well as the quality of surgical instruments. In comparison to thoracotomy, the u-VATS approach has many advantages, including fewer overall complications, shorter chest tube duration, shorter surgery time, less postoperative pain, lower chest tube drainage, less intraoperative blood loss, better cosmetic outcomes, and a lower need for pain relievers after surgery^[14]^.

## Conclusion

Bilateral pulmonary hydatidosis is not a common entity, even in endemic areas. Chest radiographs and CT scans of the chest are the mainstays of diagnosis. Surgical management is still the treatment modality of choice. A minimally invasive approach using u-VATS is safe and effective, particularly with multiple and bilateral cysts.

## Strengths and limitations

While our report successfully demonstrates the presentation and management of an atypical case of bilateral pulmonary hydatid cyst with sequential uni-portal VATS, several limitations must be acknowledged.

Primarily, as a single case report, our findings lack the generalizability of larger studies, and the absence of long-term follow-up data precludes conclusions about recurrence. Furthermore, the favorable outcome was achieved in a carefully selected patient, indicating potential selection bias. Despite these limitations, the strengths of our report lie in the documentation of this unusual presentation and the detailed illustration of a modern, minimally invasive surgical approach. The high-quality intraoperative notes clearly depict the cyst’s pathology and the key steps of the VATS procedure, providing a valuable visual reference for thoracic surgeons.

### Patient perspective

The patient was, as a nursing student, enthusiastically asking and trying to comprehend her condition and the procedures that needed to be done, and as she described it, “It was a tiring yet transformative life experience.”

## References

[1] Pump KK. Echinococcosis (hydatid disease): a review and report of a case of secondary echinococcosis. Can Med Assoc J 1963;89:73–78. 13972527 PMC1921605

[2] Khuroo MS. Hydatid disease: current status and recent advances. Ann Saudi Med 2002;22:56–64. 17259768 10.5144/0256-4947.2002.56

[3] Arinc S Kosif A Ertugrul M. Evaluation of pulmonary hydatid cyst cases. Int j surg 2009;7:192–95. 19369124 10.1016/j.ijsu.2008.11.003

[4] Kerwan A Al-Jabir A Mathew G. Revised Surgical CAse REport (SCARE) guideline: an update for the age of Artificial Intelligence. Prem J Sci 2025;10:100079.

[5] Rawat S Kumar R Raja J. Pulmonary hydatid cyst: review of literature. J Family Med Prim Care 2019;8:2774–78. 31681642 10.4103/jfmpc.jfmpc_624_19 PMC6820383

[6] Mezgar Z Khrouf M Ben Soltane H. Case of massive hydatid pulmonary embolism incidentally discovered in a 56-year-old woman with posttraumatic abdominal pain. Case Rep Pulmonol 2018;2018:7831910. 29862109 10.1155/2018/7831910 PMC5971327

[7] Almulhim AM John S. Echinococcus granulosus. In: StatPearls. Treasure Island: StatPearls Publishing; 2023. https://www.ncbi.nlm.nih.gov/books/NBK539751/30969573

[8] Arega G Kebede RA Woldeselassie HG. Bilateral large pulmonary hydatid cyst: a rare presentation in a young child from Ethiopia. Pediatric Health Med Ther 2022;13:279–82. 35983161 10.2147/PHMT.S374091 PMC9380727

[9] Messaoudi H Zayène B Ben Ismail I. Bilateral pulmonary hydatidosis associated with uncommon muscular localization. Int J Surg Case Rep 2020;76:130–33. 33035955 10.1016/j.ijscr.2020.09.070 PMC7548401

[10] Sarkar M Pathania R Jhobta A. Cystic pulmonary hydatidosis. Lung India 2016;33:179–91. 27051107 10.4103/0970-2113.177449 PMC4797438

[11] Moro P Schantz PM. Echinococcosis: a review. Int J Infect Dis 2009;13:125–33. 18938096 10.1016/j.ijid.2008.03.037

[12] Datta P Sharma B Peters NJ. Bilateral pulmonary hydatid cyst in a young child: a rare case report from North India. J Lab Physicians 2022;14:348–50. 36119419 10.1055/s-0042-1742420 PMC9473927

[13] Morar R Feldman C. Pulmonary echinococcosis. Eur Respir J 2003;21:1069–77. 12797504 10.1183/09031936.03.00108403

[14] Ahmed SK Essa RA Bapir DH. Uniportal video-assisted thoracoscopic surgery (u-VATS) for management of pulmonary hydatid cyst: a systematic review. Ann Med Surg 2022;75:103474. 10.1016/j.amsu.2022.103474 PMC8978088 35386784

